# Effect of High-Flow Nasal Cannula versus Conventional Oxygen Therapy for Patients with Thoracoscopic Lobectomy after Extubation

**DOI:** 10.1155/2017/7894631

**Published:** 2017-02-19

**Authors:** Yuetian Yu, Xiaozhe Qian, Chunyan Liu, Cheng Zhu

**Affiliations:** ^1^Department of Critical Care Medicine, Ren Ji Hospital, School of Medicine, Shanghai Jiao Tong University, Shanghai, China; ^2^Department of Thoracic Surgery, Ren Ji Hospital, School of Medicine, Shanghai Jiao Tong University, Shanghai, China; ^3^Department of Emergency Medicine, Min Hang Central Hospital, School of Medicine, Fu Dan University, Shanghai, China; ^4^Department of Emergency, Rui Jin Hospital, School of Medicine, Shanghai Jiao Tong University, Shanghai, China

## Abstract

*Objective*. To investigate whether high-flow nasal cannula (HFNC) oxygen therapy is superior to conventional oxygen therapy for reducing hypoxemia and postoperative pulmonary complications (PPC) in patients with thoracoscopic lobectomy after extubation.* Methods*. Patients with intermediate to high risk for PPC were enrolled in this study. Subjects were randomly assigned to HFNC group (HFNCG) or conventional oxygen group (COG) following extubation. Arterial blood samples were collected after extubation at 1, 2, 6, 12, 24, 48, and 72 h. Patients with postoperative hypoxemia and PPC were recorded. Adverse events were also documented.* Results*. Totally 110 patients were randomly assigned to HFNCG (*n* = 56) and COG (*n* = 54). The occurrence rate of hypoxemia in COG was twice more than that in HFNCG (29.62% versus 12.51%, *P* < 0.05) and PaO_2_, PaO_2_/FiO_2_, and SaO_2_/FiO_2_ were significantly improved in HFNCG (*P* < 0.05) in the first 72 h following extubation. Respiratory rate and incidence of reintubation as well as needing noninvasive ventilation were also decreased in HFNCG (*P* < 0.05), whereas the incidence of pneumonia and atelectasis were similar (*P* > 0.05). Adverse effects as throat and nasal pain occurred more frequently in COG.* Conclusions*. HFNC application improves oxygenation and reduces the risk of reintubation following thoracoscopic lobectomy but cannot decrease the incidence of PPC.

## 1. Introduction

Postextubation respiratory failure following major surgery is common, and a substantial proportion of the patients requires prolonged mechanical ventilation and prolonged intensive care unit (ICU) or hospital stay. Postoperative pulmonary complications (PPC) such as hypercapnia, atelectasis, and pneumonia which increase mortality are particularly attributable to adverse prognosis in patients with thorax surgery specially with lobectomy [1].

Respiratory support and oxygen therapy after tracheal extubation are of major importance to prevent hypoxemia and subsequent respiratory failure or reintubation in patients under general anaesthesia operation. Although conventional oxygen therapy via nasal prongs or a facemask can supplement oxygen administration, in some of the patients specially those who have lobectomy it is ineffective in compensating for loss in lung volume or in maintaining gas exchange [2]. High-flow nasal cannula oxygen (HFNC) mainly delivers a flow-dependent positive airway pressure and improves oxygenation by increasing end-expiratory lung volume, which can provide a maximum flow of 60 L/min [3]. It is considered to have a number of physiological advantages compared with other standard oxygen therapies, including the provision of positive end-expiratory pressure (PEEP), constant FiO_2_, and good humidification. More importantly, it can reduce the anatomical dead space [4].

A few studies comparing HFNC and conventional oxygen therapy in patients with cardiac or abdominal surgery have been published in the last five years. However, the results remained controversial due to sample heterogeneity and there was no study reported in lobectomy patients. We therefore hypothesized that HFNC treatment might be superior to conventional oxygen therapy in reducing the incidence of hypoxemia and PPC for patients with lobectomy after extubation.

## 2. Methods

### 2.1. Research Briefs

The present study was a multicenter (total of 155 ICU beds from three teaching hospitals) prospective interventional trial which was approved by the Review Board and Ethics Committee of Shanghai Jiaotong University School of Medicine (number: 2015-Clinical-Res-005). The study was unblinded and noncrossover. Informed consent was obtained for all patients, either from the patient or from the next of kin. The study took place from January 2015 to June 2016. One month before the start of this study, a standardized weaning protocol and statistics procedure were set by 9 investigators from these centers after 2 days' learning and discussion. All of the centers carefully followed this procedure during the study time.

### 2.2. Study Population

Consecutive sampling was used to recruit the patients who underwent planned thoracoscopic lobectomy because of lung tumor. Patients with intermediate to high risk for postoperative pulmonary complications (PPC) were eligible for participation. To identify such patients, the Assess Respiratory Risk in Surgical Patients in Catalonia (ARISCAT) score was used (Supplement 1 in Supplementary Material, available online at https://doi.org/10.1155/2017/7894631). ARISCAT score ≥ 26 is associated with an intermediate to high risk for PPC [5, 6]. Patients were excluded from the study if they were immunocompromised; were pregnant; converted to an open thoracotomy because of poor visualization or bleeding; or were aged <18 or >80 years or if informed consent could not be obtained.

### 2.3. Randomization, Intervention, and Weaning Protocol

All the patients eligible were transferred to ICU for postoperative monitoring and ventilator weaning at the end of the thoracoscopic lobectomy procedure. Patients were classified into two groups by random figure table following extubation. A random number sequence was generated with STATA statistical software version 12.1. HFNC oxygen therapy group (HFNCG) received a flow rate of 35 to 60 L/min and FiO_2_ was titrated (from 45% to 100%) by the treating clinician to maintain a peripheral oxygen saturation (SpO_2_) of 95% or more. The conventional oxygen therapy group (COG) received oxygen via either nasal prongs or facemask with oxygen flow titrated (from 45% to 100%) by the bedside clinician to maintain a SpO_2_ of 95% or more. HFNC oxygen therapy was delivered by the Optiflow™ system (Fisher & Paykel Healthcare Ltd, Auckland, New Zealand) using a MR850 heated humidifier and a RT202 breathing circuit. Natural air includes about 21% oxygen. If a patient is wearing a nasal cannula or a simple facemask, each additional liter/min of oxygen adds about 4 percentage points for the first 3 liters and only 3 percentage points for every liter thereafter to their FiO_2_.

Following the guideline of Difficult Airway Society Extubation Guidelines Group [7], the patients were ready for scheduled extubation after tolerating a spontaneous breathing trial in ICU. The decision to extubate was at the discretion of the treating doctors in ICU and no mandatory extubation variables were set.

### 2.4. Clinical Assessment and Outcomes

Baseline assessment included the evaluation of age, gender, body mass index (BMI), acute physiology and chronic health evaluation (APACHE) II score, ARISCAT score, baseline PaO_2_, arterial oxygen tension to inspiratory oxygen fraction ratio (PaO_2_/FiO_2_), oxygen saturation to FiO_2_ ratio (SaO_2_/FiO_2_), and PaCO_2_ before operation. Asthma, chronic obstructive pulmonary disease (COPD), and smoke history were also recorded. Lung function before operation was measured as well and the value of functional residual capacity (FRC) and forced expiratory volume in one second (FEV1)/forced vital capacity (FVC) were recorded.

The incidence of hypoxemia (defined as PaO_2_/FiO_2_ of 300 mmHg or less [8]) was recorded in the first 72 h after extubation and the differences of PaO_2_, PaO_2_/FiO_2_, SaO_2_/FiO_2_, and PaCO_2_ between the two groups were compared. Secondly, the rates of PPC like suspected pneumonia (patient receives antibiotics and meets at least one of the following criteria: new or changed sputum, new or changed lung opacities on chest X-ray when clinically indicated, tympanic temperature >38.3°C, and white blood cell (WBC) count >12 *∗* 10^9^/L in the absence of other infectious focus) and atelectasis (opacification of the lung with shift of the mediastinum, hemidiaphragm toward the affected area, and compensatory overinflation in the adjacent nonatelectatic lung) [5] were also documented. Acute hypoxemic respiratory failure was defined by one of the hypoxemic criteria (SpO_2_ < 92% while breathing at least 10 L/min oxygen, PaO_2_ < 60 mmHg on air or PaO_2_ < 80 mmHg while breathing any supplemental oxygen) and at least one of the following: severe respiratory distress with dyspnoea, accessory muscle recruitment and paradoxical abdominal or thoracic motion, respiratory rate >25 breaths/min, respiratory acidosis with pH < 7.30, and arterial carbon dioxide partial pressure (PaCO_2_) >50 mmHg [8]. Once a patient after extubation was found with acute hypoxemic respiratory failure, noninvasive ventilation (NIV) (Bipap Vision with humidification, Respironics Inc, USA) was adopted. If the symptoms of respiratory distress did not improve within 2 hours, then reintubation might be considered. The incidence of NIV requirement and reintubation were also compared.

Adverse effects related to HFNC application and oxygen therapy (air leak, throat or nasal pain, and abdominal distension) were also recorded. As the previous studies indicated that it was with high incidence of PPC within 72 h following thoracoscopic lobectomy [1, 9], the arterial blood gases were consecutively collected and checked at 1, 2, 6, 12, 24, 48, and 72 h after extubation.

### 2.5. Statistical Analysis

Review of data from the three study centers over a 3-year period (2012~2014) revealed about 30% of patients with hypoxemia who underwent thoracoscopic lobectomy after extubation. A sample size of 117 for each group provided 80% power to detect a reduction in hypoxemia from 30% to 15% (alpha = 0.05).

Statistical analysis was performed using SPSS version 19.0. Data were initially assessed for normality and were subject to log-transformation where appropriate [10]. Data between the HFNCG and COG were compared using Chi-square test for equal proportion or Fisher exact test where numbers were small with results presented as the number and percentage. Continuous variables with normal distribution were compared using Student's *t*-test and presented as means (standard deviations), whereas skewed data was compared using Wilcoxon rank-sum test and reported as medians (interquartile range). Two-way analysis of variance (ANOVA) for repeated measures with Bonferroni post hoc analysis was used for analysis of the modification of variables over time in the two groups. All analyses were performed on an intention-to-treat basis and a two-sided *P* < 0.05 was considered to be statistically significant. Figures were drawn using Graphpad prism version 6.0.

## 3. Results

### 3.1. Characteristics of the Patients

**Figure 1:**
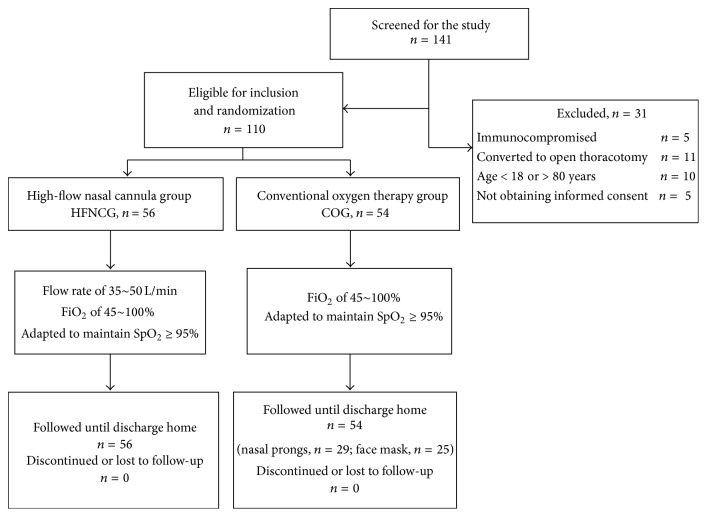
Flow chart of the study.

Over the study period, a total of 141 patients were screened and 110 eligible patients were recruited for the study. A total of 56 patients were assigned to HFNCG and 54 patients to COG. Thirty-one patients who met the exclusion criteria were excluded from the study. All patients included were followed until discharge home (Figure 1). The baseline characteristics of the 110 eligible patients are shown in Table 1. There were no significant differences between patients in two groups in all aspects (*P* > 0.05). Lung squamous cell carcinoma was the most prevalent type in both groups (57.14% versus 59.26%, *P* > 0.05).

### 3.2. Outcomes Comparison

Although lung tumor was removed by thoracoscopic surgery in a relatively less invasive way, the incidence of hypoxemia was still high during the study period due to the high risk of the patients included. The occurrence rate of hypoxemia in COG was 29.62%, two times more than that in HFNCG (12.51%), *P* < 0.05. The rate of needing NIV was still high in COG as well as the rate of reintubation (*P* < 0.05). There was no significant difference in the rate of hypercapnia, atelectasis, and suspected pneumonia (Table 2).

Because there were two different oxygen supplement strategies (via prongs or facemask) in COG, the outcomes between nasal prongs and facemask patients were also compared. It was indicated that different ways of oxygen therapy in COG did not affect the outcomes 72 h following extubation (*P* > 0.05) (Table 3). Because all subjects included in our study had intermediate to high risk for PPC, different oxygen concentrations were supplied following extubation. It revealed that hypoxemia, reintubation, and needing NIV were more likely to occur in higher oxygen concentrations patients in COG (*P* < 0.05) while there was no difference in HFNCG (*P* > 0.05) (Table 4).

We also found that there was no difference in mortality, length of ICU stay, and length of hospital stay between the two groups (*P* > 0.05). However, the total hospitalization expenditures in COG was higher than that in HFNCG (Table 5).

**Figure 2:**
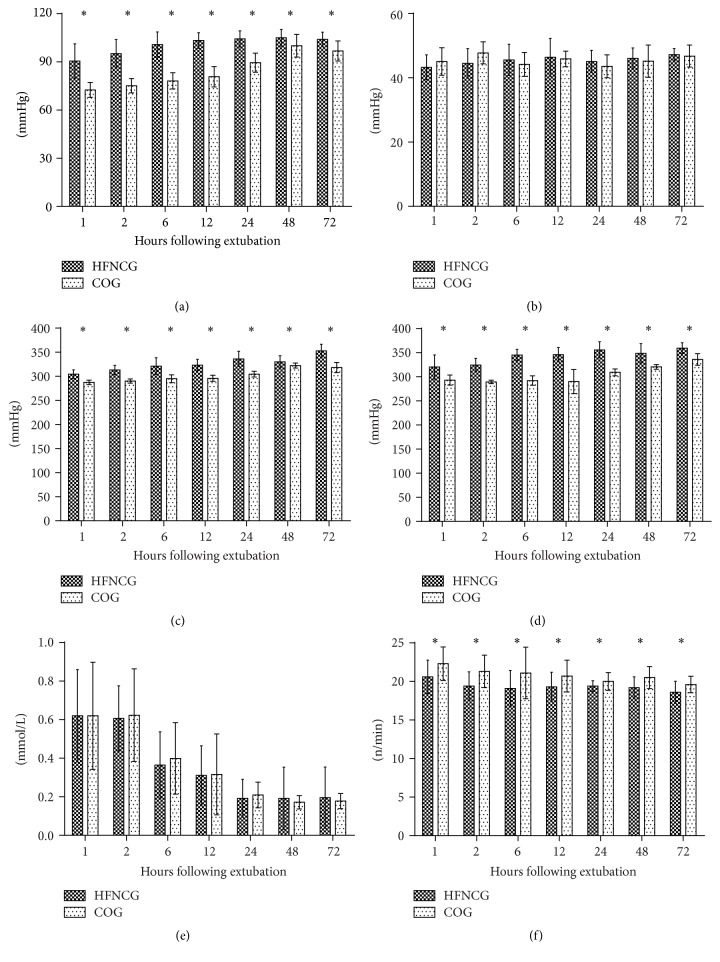
Comparison of variables between two groups in different time points. (a) Arterial PaO_2_ values; (b) arterial PaCO_2_ values; (c) the PaO_2_/fractional inspired oxygen (FiO_2_) ratio; (d) oxygen saturation/FiO_2_ ratio; (e) lactate values; (f) respiratory rate. ^*∗*^*P* < 0.05.

According to ANOVA for repeated measures, the values of PaCO_2_ and lactate were similar within the two groups throughout the entire study period in all time points (*P* > 0.05). PaO_2_ values were higher in the HFNC group at all time points, respectively (*P* < 0.05), as well as the PaO_2_/FiO_2_ ratio and SaO_2_/FiO_2_ ratio. We also found that the respiratory rate in HFNC was not that high in COG during the 72 h after extubation (Figure 2).

### 3.3. Adverse Effects Comparison

In three patients in HFNCG abdominal distension occurred during the time of oxygen therapy but the HFNC therapy could still be continued, while there was none in COG. Throat or nasal pain seems to have a high morbidity in COG (12.96%) for lack of proper humidity. No other adverse effects related to oxygen therapy, such as nasal trauma or intolerance of the therapy, need for supplemental sedation, and air leak, were found during our study period (Table 5).

## 4. Discussion

In this multicenter randomized interventional trial, we found that the application of HFNC oxygen therapy in patients with thoracoscopic lobectomy after extubation could reduce the risk of hypoxemia and reintubation as well as improve oxygenation represented by PaO_2_, PaO_2_/FiO_2_, and SaO_2_/FiO_2_. Despite extensive physiological data, there are few data on the use of HFNC in preventing the worsening of respiratory function following lobectomy. To the best of our knowledge, this study is the first randomized, controlled trial exploring the use of HFNC in adult thoracoscopic lobectomy patients.

HFNC is widely employed for patients of all age groups in several types of respiratory failure from preterm infants to adults [11] and is broadly used in ICU because of the ease of use, tolerability, and safety [3]. It has been reported to improve oxygenation after extubation in infants [12]. One clinical study indicated that HFNC might have benefit in patients with abdominal surgery [13]. However, after two years of research, they failed to identify beneficial effect of HFNC therapy after extubation in terms of the reduction of either reintubation or NIV application. Another multicenter randomized clinical trial showed that, in low risk patients, HFNC reduced the risk of reintubation within 72 hours [14]. Collectively, current literature cannot provide definitive evidence on whether HFNC treatment is superior to conventional oxygen therapy. Among the reasons, the most important factor was that patients involved in these studies were mixed populations which leaded to high heterogeneity. In our study, only patients with thoracoscopic lobectomy following extubation were included, which reduced the heterogeneity of sample and avoided misinterpreting the results.

One observational trial by Sztrymf et al. [15] demonstrated that HFNC was associated with significant reductions in respiratory rate and thoracoabdominal asynchrony and a significant improvement in arterial oxygen saturation. Our findings are consistent with this study. In the first 72 h after extubation, respiratory rate in HFNCG was lower than that in COG at any time point. The total medical costs in COG were much higher. In our opinion, relatively more NIV and reintubation in COG may help to explain the increased medical costs, since either reintubation or NIV application will prolong ICU and hospital stay. However, we could not reach the conclusion in our study. First of all, the percentage of patients with COPD or asthma was relatively low in both of the two groups (<15%), leading to a short duration of NIV usage (less than 3 days). Secondly, we acknowledged that a comparatively small sample size in our study might compromise the statistical power of the study.

In our study, the patients enrolled were those who underwent lobectomy and the occurrence of hypoxemia in HFNCG was relatively lower than that in COG, as well as the rate of needing NIV and the rate of reintubation (*P* < 0.05) which reflected the advantage of HFNC.

Although several approaches for providing supplemental oxygen have been suggested, the best option for patients with postoperation extubation remains unclear. HFNC delivers some level of continuous positive airway pressure (CPAP) via high-flow ventilation. However, the value of CPAP is unstable (from 1 to 7 cm H_2_O) because of the leak around the nasal cannula and a closed mouth of the patients cannot always be guaranteed [16]. Due to the provision of distending pressure and increase in end-expiratory lung volume, some researchers proposed that it decreased airway resistance and flushed nasopharyngeal dead space, thereby contributing to the reduced work of breathing [17, 18]. Considering the suspected induced effects of HFNC on lung volumes [19], we hypothesized that early initiation of HFNC could minimize in part lung derecruitment after extubation. Itagaki et al. reported better thoracoabdominal synchrony, measured using respiratory inductance plethysmography, in patients with mild to moderate respiratory failure when comparing high-flow nasal cannulas to standard oxygen therapy [20]. In this respect, our results support the findings of previous studies [21, 22], which suggest that HFNC oxygen therapy may favorably decrease the respiratory rate both in infants and in adult patients.

Several studies [17, 20] have demonstrated that HFNC could accelerate the elimination of CO_2_ and bronchial secretions which indicated that it might decrease the incidence of hypercapnia and pneumonia. Accordingly, we speculated that the increased flow of HFNC was able to reduce the work of breathing by flushing the nasopharyngeal space and improve CO_2_ elimination after extubation after lobectomy. However, there was no difference in the incidence of hypercapnia and pneumonia between the groups in our study which was due to few patients with severe COPD or with muscle fatigue who were included in study period.

Dry or poorly humidified medical gas may elicit patient complaints, such as dry nose, dry throat, and nasal pain, and consequent poor tolerance of oxygen therapy. Better patient comfort, a reduction in respiratory rate with a similar arterial carbon dioxide, and a higher oxygenation were reported during high-flow nasal cannula support in Roca et al.'s study [23]. However, abdominal distension which was a major complication caused by HFNC in our study is a problem that cannot be ignored. HFNC is an open ventilation system, yet it is still able to increase end-expiratory pressure. Pharyngeal pressure is affected by mouth-opening or closing, delivered flow, and size of nasal prongs. Usually, pharyngeal pressure is less than 5 cm H_2_O; however, pressure is not predictable or sustained, so we shall apply HFNC with caution in gastrointestinal surgery patients. In addition, with high flow there is an increase in the airway pressure which may make the population of our study at risk of air leak following thoracic surgery. During the study period, no patients of air leak were reported due to the pressure generated by HFNC being relatively low.

### 4.1. Study Limitation

We acknowledge that there are limitations in the study. We calculated the sample size by using a historical review of extubation data from 2012 to 2014 before the study initiated. The sample size expected in each group was 117. However, during our 18-month (January 2015 to June 2016) study period, the patients eligible were not as much as we expected because the majority of patients with lobectomy were at low risk which might have been attributable to overall improvements in perioperative surgical care. This might lead to compromised statistical power to detect a significant difference between groups in the primary outcome. In addition, postoperative changes in pulmonary function were not recorded and compared. Anyway, our study did show a clear clinical benefit of high-flow nasal cannula oxygen therapy.

### 4.2. Conclusion

HFNC application improves oxygenation and reduces the risk of reintubation. It should be used as an alternative to the conventional oxygen therapy especially for patients receiving thoracoscopic lobectomy following extubation.

## Supplementary Material

The Assess Respiratory Risk in Surgical Patients in Catalonia (ARISCAT) index includes seven independent risk factors (four patient-related risk factors and three related to the surgical procedure, accounting for 55% and 45% of the score respectively). The index can be used to assess individual risk of postoperative pulmonary complications and focus on investigating patients with intermediate to high risk of complications with the most relevant cut point being the risk score of 26.

## Figures and Tables

**Table 1:** Demographic characteristics of the patients who participated in the study (mean ± SD).

Characteristics	HFNCG (*n* = 56)	COG (*n* = 54)	*P*
Age, yrs	56.31 ± 7.03	55.82 ± 7.92	0.732
Male gender, *n* (%)	30 (53.57)	28 (51.85)	1.000
BMI, kg/m^2^	26.32 ± 4.73	25.19 ± 5.02	0.226
APACHE II	26.32 ± 4.73	25.19 ± 5.02	0.226
ARISCAT	31.12 ± 3.74	32.36 ± 3.08	0.071
COPD, *n* (%)	8 (14.29)	7 (12.96)	0.840
Asthma, *n* (%)	5 (8.93)	4 (7.41)	1.000
Smoking history, *n* (%)	12 (21.43)	8 (14.81)	0.369
Hemoglobin, g/L	108.29 ± 17.31	105.43 ± 22.06	0.450
Lactate, mmol/L	0.32 ± 0.07	0.33 ± 0.06	0.424
Respiratory, /min	18.43 ± 3.45	17.98 ± 3.87	0.521
PaO_2_, mmHg	95.37 ± 12.42	92.59 ± 18.49	0.355
PaCO_2_, mmHg	41.73 ± 6.33	43.52 ± 4.93	0.102
PaO_2_/FiO_2_, mmHg	350.35 ± 33.87	340.98 ± 40.65	0.191
SaO_2_/FiO_2_	210.37 ± 52.77	222.51 ± 48.65	0.213
FRC, L	2.08 ± 0.32	2.12 ± 0.41	0.567
FEV1/FVC, %	78.63 ± 11.52	75.52 ± 13.45	0.195
Postsurgical ventilation durations, h	2.13 ± 0.43	2.18 ± 0.32	0.492

**Table 2:** Occurrence rates for outcomes in COG compared with HFNCG 72 h following extubation, *n* (%).

Characteristics	HFNCG (*n* = 56)	COG (*n* = 54)	*P*
Hypoxemia	7 (12.50)	16 (29.63)	0.027
Hypercapnia	3 (5.36)	8 (14.81)	0.121
Reintubation	0 (0)	5 (9.26)	0.026
Needing NIV	2 (3.57)	9 (16.67)	0.027
Atelectasis	2 (3.57)	5 (9.26)	0.266
Suspected pneumonia	2 (3.57)	2 (3.70)	1.000
Throat or nasal pain	1 (1.79)	7 (12.96)	0.030
Abdominal distension	3 (5.36)	0 (0)	0.243
Air leak	0 (0)	0 (0)	1.000

**Table 3:** Occurrence rates for outcomes in nasal prongs patients compared with facemask patients 72 h following extubation, *n* (%).

Characteristics	Nasal prongs (*n* = 29)	Facemask (*n* = 25)	*P*
Hypoxemia	9 (31.03)	7 (28.00)	0.808
Hypercapnia	4 (13.79)	4 (16.00)	1.000
Reintubation	3 (10.34)	2 (8.00)	1.000
Needing NIV	4 (13.79)	5 (20.00)	0.718
Atelectasis	2 (6.89)	3 (12.00)	0.653
Suspected pneumonia	1 (3.45)	1 (4.00)	1.000
Throat or nasal pain	3 (10.34)	4 (16.00)	0.692
Abdominal distension	0 (0)	0 (0)	1.000
Air leak	0 (0)	0 (0)	1.000

**Table 4:** Occurrence rates for outcomes in HFNC patients compared with COG patients with different oxygen concentrations 72 h following extubation, *n* (%).

Characteristics	HFNCG (*n* = 56)			*P*	COG (*n* = 54)			*P*
	FiO_2_ (45~60%)	FiO_2_ (60~80%)	FiO_2_ (80~100%)		FiO_2_ (45~60%)	FiO_2_ (60~80%)	FiO_2_ (80~100%)	
	(*n* = 18)	(*n* = 22)	(*n* = 16)		(*n* = 17)	(*n* = 21)	(*n* = 16)	
Hypoxemia	2 (11.11)	2 (9.09)	3 (18.75)	0.658	2 (11.76)	5 (23.81)	9 (56.25)	0.015
Hypercapnia	1 (5.56)	1 (4.55)	1 (6.25)	0.973	2 (11.76)	4 (19.05)	2 (12.50)	0.782
Reintubation	0 (0)	0 (0)	0 (0)	1.000	0 (0)	1 (4.76)	4 (25.00)	0.031
Needing NIV	0 (0)	0 (0)	2 (12.5)	0.075	0 (0)	3 (14.29)	6 (37.50)	0.014
Atelectasis	1 (5.56)	0 (0)	1 (6.25)	0.508	1 (5.88)	2 (9.52)	2 (12.50)	0.806
Suspected pneumonia	0 (0)	1 (4.55)	1 (6.25)	0.588	0 (0)	1 (4.76)	1 (6.25)	0.603
Throat or nasal pain	0 (0)	1 (4.55)	0 (0)	0.455	0 (0)	2 (9.52)	5 (31.25)	0.024
Abdominal distension	0 (0)	0 (0)	3 (18.75)	0.019	0 (0)	0 (0)	0 (0)	1.000
Air leak	0 (0)	0 (0)	0 (0)	1.000	0 (0)	0 (0)	0 (0)	1.000

**Table 5:** Comparison of hospitalizations between two groups (mean ± SD).

Characteristics	HFNCG (*n* = 56)	COG (*n* = 54)	*P*
Mortality	0	0	1.000
Length of ICU stay, days	3.72 ± 0.56	3.64 ± 0.83	0.553
Length of hospital stay, days	7.41 ± 0.82	7.54 ± 0.91	0.433
Total hospitalization expenditures, $	11522.65 ± 762.45	12219.73 ± 1028.66	0.001

## Abbreviations

APACHE: Acute physiology and chronic health evaluation
ARISCAT: Assess Respiratory Risk in Surgical Patients in Catalonia
BMI: Body mass index
COPD: Chronic obstructive pulmonary disease
CPAP: Continuous positive airway pressure
FEV1: Forced expiratory volume in one second
FRC: Functional residual capacity
FVC: Forced vital capacity
HFNC: High-flow nasal cannula oxygen
ICU: Intensive care unit
NIV: Noninvasive ventilation
PEEP: Positive end-expiratory pressure
PPC: Postoperative pulmonary complications.
