# Supplementary material for: Effect of High-Flow Nasal Cannula versus Conventional Oxygen Therapy for Patients with Thoracoscopic Lobectomy after Extubation
Source: Can Respir J. 2017 Feb 19;2017:7894631. doi: 10.1155/2017/7894631 (PMC5337350; doi:10.1155/2017/7894631)
Supplement: Supplementary file 1 — The Assess Respiratory Risk in Surgical Patients in Catalonia (ARISCAT) index includes seven independent risk factors (four patient-related risk factors and three related to the surgical procedure, accounting for 55% and 45% of the score respectively). The index can be used to assess individual risk of postoperative pulmonary complications and focus on investigating patients with intermediate to high risk of complications with the most relevant cut point being the risk score of 26. [file 7894631.f1.pdf]

Supplement 1. The Seven ARISCAT Risk Predictors and Points Assigned

Item	Score
**Age (yr)**	
≤50	0
51–80	3
>80	16
**Preoperative Spo2**	
≥96%	0
91–95%	8
≤90%	24
**Respiratory infection in the last month**	
No	0
Yes	17
**Preoperative anemia (Hb ≤10 g/dl)**	
No	0
Yes	11
**Surgical incision**	
Peripheral	0
Upper abdominal	15
Intrathoracic	24
**Duration of surgery (h)**	
<2	0
2–3	16
>3	23
**Emergency procedure**	
No	0
Yes	8

Three levels of risk were indicated by the following cutoffs: <26 points, low risk; 26–44 points, moderate risk; and ≥45 points, high risk.

ARISCAT = Assess Respiratory Risk in Surgical Patients in Catalonia; Hb = hemoglobin; Spo2 = arterial oxyhemoglobin saturation by pulse oximetry.
